# Effect of hemostatic agent on marginal gaps of class V giomer restorations

**DOI:** 10.4317/jced.53704

**Published:** 2017-05-01

**Authors:** Soodabeh Kimyai, Fatemeh Pournaghi-Azar, Narmin Mohammadi, Mahdieh Babri

**Affiliations:** 1 Dental and Periodontal Research Center, Faculty of Dentistry, Tabriz University of Medical Sciences, Tabriz, Iran; 2 Professor, Department of Operative Dentistry, Faculty of Dentistry, Tabriz University of Medical Sciences, Tabriz, Iran; 3 Assistant Professor, Department of Operative Dentistry, Faculty of Dentistry, Tabriz University of Medical Sciences, Tabriz, Iran; 4 Under graduate student, Department of Operative Dentistry, Faculty of Dentistry, Tabriz University of Medical Sciences, Tabriz, Iran

## Abstract

**Background:**

Contamination of dentin with hemostatic agents might exert a deleterious effect on adhesive procedures on dentin. The present study was undertaken to investigate the effect of aluminum chloride hemostatic agent on marginal gaps in Cl V giomer restorations.

**Material and Methods:**

Fifty sound bovine permanent incisors were selected for the purpose of this *in vitro* study and Cl V cavities were prepared on their buccal surfaces; the gingival margins of the cavities were placed in dentin. The tooth samples were randomly assigned to two groups (n=25). The samples in groups 1 and 2 underwent a restorative procedure without and with the application of aluminum chloride hemostatic agent in the cavity, respectively, before application of the adhesive. BeautiBond one-step self-etch adhesive and Beautifil II giomer restorative material were used for the restoration of the cavities in both groups. The samples were thermocycled and sectioned, followed by measuring the gap sizes at gingival margins in µm under a stereomicroscope. The marginal gaps were compared with Mann-Whitney U test. Statistical significance was set at *P*<0.05.

**Results:**

The results showed significant differences in the mean marginal gaps between the two groups under study (*P*<0.001); the mean marginal gaps were higher in group 2 (with hemostatic agent) compared to those in group 1 (without hemostatic agent) (*P*<0.0005).

**Conclusions:**

Contamination with aluminum chloride hemostatic agent in giomer restorations gave rise to higher gingival margin gaps.

** Key words:**Dental adhesives, giomer restorative material, hemostatic agent, marginal adaptation.

## Introduction

One of the most important and effective factors in the success of composite resin restorations is proper isolation and management of contamination resulting from blood and gingival crevicular fluid. Surface contaminations can interfere with the adhesion of composite resins to dentin. The quality of the bond between composite resins and dentin entails a proper interaction between the resin and collagen fibers of dentin that are free of mineral agents (1,2). It has been reported that the gingival crevicular fluid and blood can occlude the dentinal tubules and prevent penetration of resin into the collagen fibers, interfering with proper bonding, finally decreasing the bond strength (3-5).

Disturbance of the dentinal seal and the presence of gaps in restorations can result in microleakage. The persistence of microleakage can result in tooth sensitivity, recurrent caries, irritation of the pulp and failure of treatment (6). Therefore, in order to improve the quality of restoration and increase their longevity, attempts should be made to control bleeding and gingival crevicular fluid; a technique to achieve this is the use of hemostatic agents (7-9). Aluminum chloride is one of the hemostatic agents that is used to this end. However, a study showed that this agent interferes with the bonding of self-etch adhesives to dentin due to its acidic nature that results in the removal of the smear layer (10). Different studies have reported varying results in this respect (10-14). Studies by Khoroushi *et al.* and Ebrahimi *et al.* showed that hemostatic agents decrease the bond strength of composite resins to dentin (11,12). In addition, a study by Mohammadi *et al.* showed that the hemostatic agent resulted in an increase in the microleakage of Cl V composite resin restorations (13). However, in a study by Xu *et al.*, use of hemostatic agents had no effect on the shear bond strength of composite resin to dentin (14). In addition, in a study by Kuphasuk *et al.* on the bond strength of two types of adhesive agents contaminated with a hemostatic agent, the hemostatic agent resulted in a decrease in the bond strength of the self-etch adhesive resin; however, there was no significant difference in the bond strength of the total-etch adhesive between the contaminated dentin and normal dentin (10).

Recently, a new group of composite resins, giomers, have been introduced for direct adhesive restorations that have the advantages of glass-ionomers (release of fluoride and the capacity to recharge) and composite resins (easy polishability, esthetics and biocompatibility) together. In relation to photoactivation, giomers are similar to composite resins and require the use of a bonding procedure to bond to tooth structure. Giomers can be used for the restoration of Cl I to Cl V cavities, cervical erosions and root surface caries (15-19).

Considering the fact that hemostatic agents can induce changes on the dentin surface, affecting the bonding of restorations (20) and since no studies to date have evaluated the effect of hemostatic agents on the marginal gaps of giomer restorations and the results of studies on composite resins and other adhesives cannot be generalized to giomer restorations due to differences in their chemical compositions (21), the present in vitro study was undertaken to evaluate the effect of contamination with aluminum chloride hemostatic agent on the marginal gaps of Cl V giomer restorations.

## Material and Methods

Fifty sound permanent bovine mandibular incisors were selected for the purpose of this *in vitro* study. The Regional Medical Research Ethics Committee approved the protocol of this study. The selected tooth samples exhibited no abrasions, cracks and morphological defects when they were examined visually and under a stereomicroscope (Nikon, Tokyo, Japan). All the tooth samples were used within one month after they were extracted. The samples were immersed in a 0.5% chloramine-T trihydrate bacteriostatic/bacteiocidal solution (Merck KGaA, Darmstadt, Germany) for a week, followed by storage in distilled water within a refrigerator at a temperature of 4ºC. The storage medium was refreshed on a regular basis. Twenty-four hours before the initiation of the experimental procedures, the tooth samples were conditioned in distilled water at a temperature of 23±2ºC, followed by being randomly assigned to two groups of 25 and processed as follows:

In group 1, the buccal surfaces underwent a Cl V cavity preparation procedure with the use of a high-speed handpiece and a cylindrical diamond bur (Diatech Dental AG, Swiss Dental Instruments, CH-9435 Heerbrugg); the cavities measured 3×3 mm occlusogingivally and mesiodistally and 2 mm in depth. The occlusal and gingival walls of the cavities were placed 1.5 mm coronal and apical to the CEJ, respectively. No beveling was carried out on the cavity margins; therefore, they were butt-jointed. Care was exercised to prevent dehydration of tooth surfaces during the preparation procedures. BeautiBond (Shofu Inc., Kyoto, Japan) one-step self-etch adhesive was applied on all the cavity walls based on manufacturer’s instructions, followed by light-curing with an Astralis 7 halogen light-curing unit (Ivoclar Vivadent, Schaan, Liechtenstein). The adhesive was light-cured for 10 seconds based on manufacturer’s instructions. Using A2 shade of Beautifil II giomer restorative material (Shofu Inc., Kyoto, Japan) the cavities were restored with the incremental technique (two layers separately, measuring 1 mm in thickness). Each restorative material layer was light-cured for 20 seconds at a light intensity of 400 mW/cm2, with the light conducting tip perpendicular and very close to the material surface. Post-curing was implemented for 60 seconds at a light intensity of 700 mW/cm2.

In group 2, all the procedures above, described for group 1, were repeated except that prior to the application of the adhesive resin the gingival margins were contaminated with aluminum chloride which is a hemostatic agent (Hemopare, Maquira Industry Dental Products Ltd, Brazil), with a mini-brush. Then the margins were irrigated with water from a water spray for 30 seconds after 5 minutes (based on manufacturer’s instructions) and dried with an air syringe.

**Figure 1:**
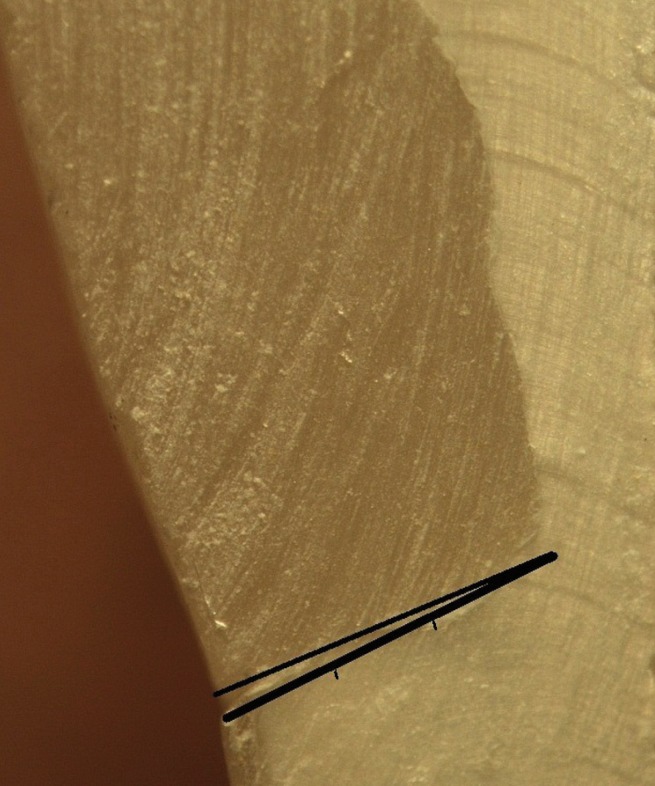
The schematic representation showing the method to measure marginal gap. Gap width was measured at three parts (outer, middle, and inner parts) of the gingival margin.

One operator completed all the restorative procedures. At the end of restorative procedures, the specimens were finished and polished using diamond finishing burs (Diamont Gmbh, D&Z, Berlin, Germany) and polishing disks (Sof-Lex, 3M ESPE, Dental Products, St. Paul, MN, USA), followed by immersion in distilled water and incubation at 37ºC for 24 hours. In order to mimic the conditions prevailing in oral cavity, the tooth samples underwent a thermocycling procedure at 5±2/55±2ºC, consisting of 500 rounds with a dwell time of 30 seconds and a transfer time of 10 seconds. Finally, the samples were sectioned in a buccolingual direction at the middle of the restorations, with a diamond disk (Diamont Gmbh, D&Z, Berlin, Germany), followed by measuring the gingival marginal gaps at ×40 under a stereomicroscope (Nikon, Tokyo, Japan) (22). Selected areas underwent digital photography with a DS-L2 control unit (Nikon, Tokyo, Japan) so that the gap sizes could be determined. The gaps were measured with the use of the built-in software by drawing a line tangential to the tooth-side vector in order to determine the distance between the points on the vector that was located on the restoration side and the line above. The measurement procedures were repeated at three locations: the outer, middle and inner portions of gingival margins. Figure 1 shows the method for evaluation of the gaps at the gingival margin. The means were calculated for these three marginal gap sizes in micrometers in both study groups. Data on marginal gaps in the two groups were evaluated with Mann-Whitney U test because data was not distributed normally based on Kolmogorov-Smirnov test results (*P*<0.001) and inequality of variances based on Leven’s test (*P*=0.001) using SPSS 20.0. Statistical significance was set at *P*<0.05.

## Results

**Figure 2:**
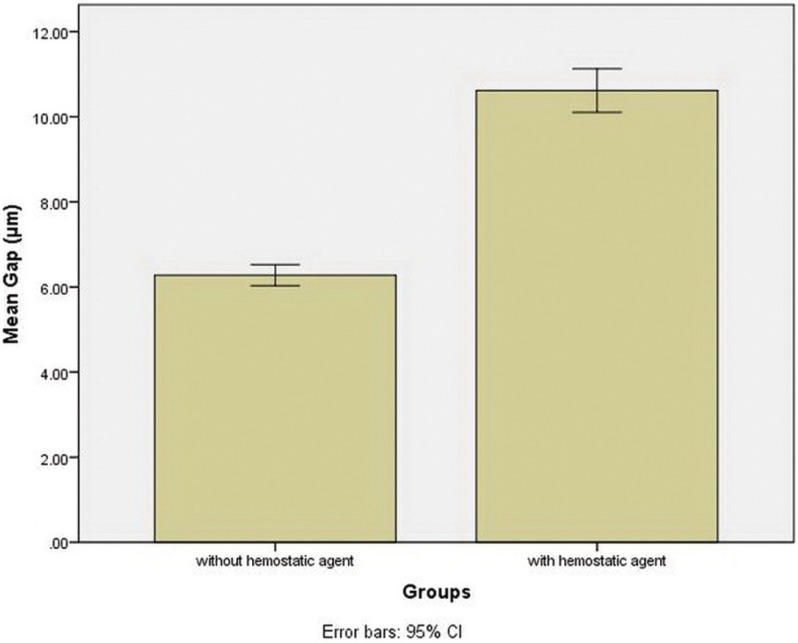
Bar graph of the mean marginal gaps in the two study groups.

Figure 2 shows the bar graph of mean marginal gap sizes in both study groups. In groups 1 and 2 the means and standard deviations of marginal gaps were 6.27±0.56 and 10.61±1.23 µm, respectively.

Evaluation of the results of Mann-Whitney U test showed a significant difference in the mean marginal gaps between the two groups (*P*<0.001), with a higher mean of marginal gaps in group 2 (with the use of the hemostatic agent) compared to group 1 (without the use of hemostatic agent) (*P*<0.0005).

## Discussion

It is of vital importance to eliminate gingival bleeding or contamination by gingival crevicular fluid to achieve a durable restoration (12). Aluminum chloride is one of the most commonly used hemostatic agents to control bleeding and gingival crevicular fluid (13). Therefore, in the present study the effect of contamination with aluminum chloride hemostatic agent on gingival margin gaps of Cl V giomer restorations was evaluated.

The results of the present study showed that in the group receiving the hemostatic agent there were more marginal gaps compared to the group without the hemostatic agent. It appears this can be attributed to the presence of contamination with aluminum chloride on the dentin surface and its interference with the bonding process. It was shown in a study by Kuphasuk *et al.* that even irrigation with water did not result in complete elimination of contamination with aluminum chloride (10) and electron scanning micrographs showed remnants of aluminum chloride on the dentin surface (3,12). However, in a study by O’Keefe *et al.*, irrigation with water after the application of aluminum chloride hemostatic agent resulted in an increase in the bond strength of a self-etch adhesive compared to the situation in which irrigation was not carried out (23).

In addition, it has been reported that self-etch adhesives cannot completely eliminate the hemostatic agent due to their higher pH and lower acid etching properties and they cannot completely penetrate into the deeper areas of dentin. Therefore, their application subsequent to the use of aluminum chloride hemostatic agent resulted in a decrease in dentin bond strength (1,3). Since the adhesive used in the present study (BeautiBond) was a self-etch adhesive with weak acidity (pH=2.4) (24), it appears it acted in a similar manner on the dentin contaminated with the hemostatic agent, resulting in an increase in marginal gaps. Another reason for an increase in marginal gap in the group with hemostatic agent might be the acidic properties of the hemostatic agents (pH=0.7-3). Due to their acidic nature, these materials dissolve the smear layer and occlude the dentinal tubules, forming an amorphous or granular layer on the dentin surface, which interferes with the bonding process (1,10). The adhesive used in the present study (BeautiBond) is a self-etch adhesive ad its mechanism of adhesion depends on the modification of the smear layer and the exposed collagen network by the self-etch primer in the adhesive system. Since a 5-minute application of aluminum chloride results in complete elimination of the smear layer and the peritubular dentin (3,10), it appears the absence of the smear layer interferes with the bonding of BeautiBond adhesive to dentin, which might be another reason for the formation of more gaps in the group in which the hemostatic agent was used.

In this context, a similar finding was reported in a previous study and it was shown that contamination with aluminum chloride hemostatic agent resulted in a decrease in bond strength to dentin (10). It was shown in another study that use of aluminum chloride hemostatic agent resulted in an increase in dentin margin microleakage in Cl V composite resin restorations with the use of a one-step self-etch adhesive (13). In addition, Ebrahimi *et al.* reported that contamination with iron sulfate hemostatic agent resulted in a decrease in dentin bond strength in a self-etch adhesive (12). In another study, contamination with hemostatic agents (Viscostat Clear, Viscostat, trichloroacetic acid) resulted in a decrease in dentin bond strength (11).

Contrary to the results of the present study, in a study by Kuphasuk *et al.* (10), use of aluminum chloride hemostatic agent did not result in a decrease in dentin bond strength of a total-etch adhesive. It appears such a difference can be attributed to differences in the adhesives used and complete elimination of the hemostatic agents due to acid etching in the total-etch system.

In a study by Harnirattisai *et al.* there were no differences in the dentin content of aluminum chloride residues in contaminated and non-contaminated dentin after acid etching (25).

Xu *et al.* showed that contamination with aluminum chloride, iron sulfate and aluminum sulfate hemostatic agents resulted in a negative effect on the dentin bond of a total-etch adhesive (14). In addition, it was shown in another study that use of 22% aluminum chloride hemostatic agent and 20% iron sulfate had no effect on the dentin bond strength of a total-etch adhesive (20). It appears the differences in the results might be attributed to differences in the hemostatic agents used and their different mechanisms of action on dentin and also the type of the adhesive used in the present study and previous studies (14,20).

In addition, the duration of the application of hemostatic agent can be considered another reason for differences in the results between the present study and the studies above (14,20) because in the two previous studies mentioned above the hemostatic agent was applied for one minute, while in the present study it was applied for 5 minutes. It appears the negative effect of the hemostatic agent on the bonding process and adhesion of the resin-based materials increases with an increase in the presence of the hemostatic agent on the dentin surface.

A study showed that use of EDTA and phosphoric acid on the dentin surface contaminated with aluminum chloride hemostatic agent can eliminate contamination resulting from the hemostatic agent. However, contrary to EDTA, phosphoric acid was unable to increase the dentin bond strength up to that in the control group (without contamination with the hemostatic agent) (26). It is suggested that future studies evaluate the marginal gaps of adhesive restorative materials with the use of different agents to eliminate contamination resulting from hemostatics, such as EDTA. It is also proposed that the ultra-structure of the tooth-restorative material interface be assessed.
